# Symptoms and Health Outcomes Among Survivors of COVID-19 Infection 1 Year After Discharge From Hospitals in Wuhan, China

**DOI:** 10.1001/jamanetworkopen.2021.27403

**Published:** 2021-09-29

**Authors:** Xue Zhang, Fang Wang, Ye Shen, Xiaohua Zhang, Yuan Cen, Bin Wang, Songtao Zhao, Yi Zhou, Baoman Hu, Man Wang, Yuhui Liu, Hongming Miao, Paul Jones, Xiangyu Ma, Yong He, Guoqiang Cao, Lixia Cheng, Li Li

**Affiliations:** 1 Department of Respiratory Medicine, Daping Hospital, Third Military Medical University (Army Medical University), Chongqing, China; 2 Department of General Surgery, Daping Hospital, Third Military Medical University (Army Medical University), Chongqing, China; 3 Wuhan Huoshenshan Hospital, Wuhan, China; 4 Department of Orthopedics, Daping Hospital, Third Military Medical University (Army Medical University), Chongqing, China; 5 Pulmonary and critical care medicine center, Chinese PLA Respiratory Disease Institute, Xinqiao Hospital, Third Military Medical University (Army Medical University), Chongqing, China; 6 Institute of Infectious Diseases, Southwest Hospital, Third Military Medical University (Army Medical University), Chongqing, China; 7 Institute of Cancer, Xinqiao Hospital, Third Military Medical University (Army Medical University), Chongqing, China; 8 Department of Neurology and Centre for Clinical Neuroscience, Daping Hospital, Third Military Medical University (Army Medical University), Chongqing, China; 9 Department of Biochemistry and Molecular Biology, Third Military Medical University (Army Medical University), Chongqing, China; 10 Institute of Infection and Immunology, University of London, London, United Kingdom; 11 Department of Epidemiology, College of Preventive Medicine, Third Military Medical University (Army Medical University), Chongqing, China; 12 Department of Medical and Research Management, Daping Hospital, Third Military Medical University (Army Medical University), Chongqing, China; 13 Wuhan Taikang Tongji Hospital, Wuhan, China

## Abstract

**Importance:**

The long-term health outcomes and symptom burden of COVID-19 remain largely unclear.

**Objective:**

To evaluate health outcomes of COVID-19 survivors 1 year after hospital discharge and to identify associated risk factors.

**Design, Setting, and Participants:**

This retrospective, multicenter cohort study was conducted at 2 designated hospitals, Huoshenshan Hospital and Taikang Tongji Hospital, both in Wuhan, China. All adult patients with COVID-19 discharged between February 12 and April 10, 2020, were screened for eligibility. Of a consecutive sample of 3988 discharged patients, 1555 were excluded (796 declined to participate and 759 were unable to be contacted) and the remaining 2433 patients were enrolled. All patients were interviewed via telephone from March 1 to March 20, 2021. Statistical analysis was performed from March 28 to April 18, 2021.

**Exposures:**

COVID-19.

**Main Outcomes and Measures:**

All patients participated in telephone interviews using a series of questionnaires for evaluation of symptoms, along with a chronic obstructive pulmonary disease (COPD) assessment test (CAT). Logistic regression models were used to evaluate risk factors for fatigue, dyspnea, symptom burden, or higher CAT scores.

**Results:**

Of 2433 patients at 1-year follow-up, 1205 (49.5%) were men and 680 (27.9%) were categorized into the severe disease group as defined by the World Health Organization guideline; the median (IQR) age was 60.0 (49.0-68.0) years. In total, 1095 patients (45.0%) reported at least 1 symptom. The most common symptoms included fatigue, sweating, chest tightness, anxiety, and myalgia. Older age (odds ratio [OR], 1.02; 95% CI, 1.01-1.02; *P* < .001), female sex (OR, 1.27; 95% CI, 1.06-1.52; *P* = .008), and severe disease during hospital stay (OR, 1.43; 95% CI, 1.18-1.74; *P* < .001) were associated with higher risks of fatigue. Older age (OR, 1.02; 95% CI, 1.01-1.03; *P* < .001) and severe disease (OR, 1.51; 95% CI, 1.14-1.99; *P* = .004) were associated with higher risks of having at least 3 symptoms. The median (IQR) CAT score was 2 (0-4), and a total of 161 patients (6.6%) had a CAT score of at least 10. Severe disease (OR, 1.84; 95% CI, 1.31-2.58; *P* < .001) and coexisting cerebrovascular diseases (OR, 1.95; 95% CI, 1.07-3.54; *P* = .03) were independent risk factors for CAT scores of at least 10.

**Conclusions and Relevance:**

This study found that patients with COVID-19 with severe disease during hospitalization had more postinfection symptoms and higher CAT scores.

## Key Points

**Question:**

What are the long-term health outcomes associated with COVID-19 infection 1 year after hospital discharge?

**Findings:**

In this cohort study of 2433 patients who had been hospitalized with COVID-19, the most common symptoms at 1 year after discharge were fatigue, sweating, chest tightness, anxiety, and myalgia. Patients with severe disease had more postinfection symptoms and higher chronic obstructive pulmonary disease assessment test scores.

**Meaning:**

This study reported prolonged symptoms of COVID-19 and found that severe disease during hospitalization was a risk factor for more symptoms and higher chronic obstructive pulmonary disease assessment test scores.

## Teaser

This cohort study investigates 1-year health outcomes and their associated risk factors among COVID-19 survivors in Wuhan, China.

## Introduction

COVID-19 has spread rapidly into a global pandemic ever since the initial reports in December 2019 in China, which has caused millions of deaths.^1^ The disease affects multiple systems of the body in the acute phase, represented by acute pneumonia.^2^ With the increasing number of patients recovered, postinfection health consequences have been recognized.^3,4,5^ The first survivors in Wuhan, China, have now lived for 1 year, which provides an opportunity to address the long-term sequelae of COVID-19 in a large population sample.

Several studies have reported that COVID-19 survivors might have persistent symptoms, impaired lung function, and chest imaging abnormalities.^5,6,7,8,9^ At 6 months after symptom onset, 76% of patients reported at least 1 symptom, the most common being fatigue, muscle weakness, and sleep difficulties.^10^ In a prospective cohort study of 83 patients with COVID-19, dyspnea scores and exercise capacity improved at 1 year after hospital discharge, whereas radiological changes persisted in 20 (24%) patients.^11^ In another study of COVID-19 survivors 1 year after hospital discharge, post–COVID-19 symptoms included fatigue, dyspnea, chest pain, and cough.^12^ There are currently no agreed-upon measures to assess the symptom burden of COVID-19. However, the Chronic Obstructive Pulmonary Disease (COPD) Assessment Test (CAT), an 8-item questionnaire designed to quantify health status impairment in COPD patients,^13^ demonstrated a high CAT score in patients with COVID-19 in the weeks following their admission.^14^ However, the long-term symptom burden and health outcomes remain largely unknown. This study aimed to determine the long-term health outcomes associated with COVID-19 in a group of patients discharged from 2 designated hospitals of Wuhan one year after discharge.

## Methods

This cohort study was approved by the Ethics Committee of the Daping Hospital of Army Medical University and registered at the Chinese Clinical Trial Registry (ChiCTR2100045964). Verbal informed consent was obtained from all participants or their legal guardians prior to the survey. The study followed the Strengthening the Reporting of Observational Studies in Epidemiology (STROBE) reporting guideline.

### Study Design and Patients

All adult patients with laboratory-confirmed COVID-19 who were discharged from Huoshenshan Hospital and Taikang Tongji Hospital (both in Wuhan, China) between February 12 and April 10, 2020, were screened for eligibility. The follow-up study was conducted from March 1 to March 20, 2021. All discharged patients met uniform discharge criteria according to the Chinese clinical guidance for COVID-19 pneumonia diagnosis and treatment issued by the National Health Commission.^15^ The exclusion criteria included (1) those who declined to participate and (2) those unable to be contacted.

### Procedures and Data Acquisition

All patients were contacted in the order of their discharge date as documented in the medical record. Patients were interviewed via telephone by trained physicians using a series of questionnaires, including a self-reported symptom questionnaire, and CAT, as shown in eTable 1 in the Supplement. A CAT score of at least 10 is recommended as the threshold for maintenance treatment in COPD,^16^ which was based on a modeling study of the association between CAT scores and the daily life and well-being patients with COPD.^17^ The symptom questionnaire was based on symptoms that had been reported by patients during hospitalization, which was described in an earlier study.^18^ These symptoms included fever, cough, fatigue, anorexia, shortness of breath, chest tightness, myalgia, expectoration, dyspnea, diarrhea, sore throat, nausea, vomiting, headache, chill, dizziness, and hemoptysis. Patients were asked to report any persistent or newly occurring symptoms. The patients' current symptoms were carefully distinguished from those of their preillness state or other underlying diseases not related to COVID-19.

Clinical data for patients during hospitalization were retrieved from electronic medical records, including demographic characteristics (self-reported race, age, sex, and cigarette smoking) and clinical characteristics (self-reported comorbidities, symptoms, and chest images). The disease severity was defined by World Health Organization guideline for COVID 19.^19^ Severe pneumonia refers to fever or suspected respiratory infection, plus 1 of the following: respiratory rate greater than 30 breaths per minute; severe respiratory distress; or oxygen saturation as measured by pulse oximetry (SpO_2_) less than or equal to 93% on room air.^19^ We double-entered and validated all data using EpiData software version 3.1 (EpiData Association).

### Statistical Analysis

Continuous variables were described as median with IQR, followed by Mann-Whitney *U* test. Categorical variables were presented as absolute values along with percentages and compared using the Pearson χ^2^ test or Fisher exact test. We compared the clinical characteristics between enrolled patients and those who were lost to follow-up. To test for the risk of bias due to the patients who were lost to follow-up, 1:1 propensity score–matching between the subpopulation lost to follow-up (n = 1555) and the enrolled subpopulation (n = 2433) was carried out. To explore factors associated with the risk of fatigue, dyspnea, occurrence of at least 3 symptoms, and CAT scores of at least 10, univariate logistic regression analysis was used to identify potential risk factors with *P* < .10. These were then used in a stepwise (forward:LR) selection process in multivariate logistic regression model. All tests were 2-sided, and *P* < .05 was considered statistically significant. All statistical analyses were performed with the use of R software version 3.6.2 (R Project for Statistical Computing) from March 28 to April 18, 2021.

## Results

### Patient Characteristics

**Figure 1.**
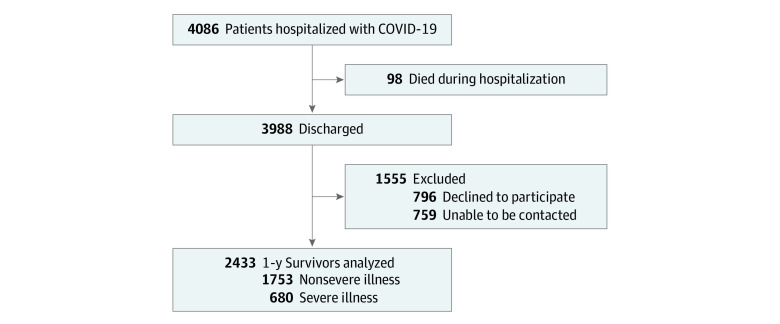
Study Flowchart

A total of 3988 COVID-19 survivors were screened for eligibility. In total, 1555 patients were excluded (796 declined to participate and 759 were unable to be contacted) and the remaining 2433 patients were enrolled for the questionnaire phone interview (Figure 1). Among the patients who were enrolled, 1205 (49.5%) were men, 1228 (50.5%) were women, and 680 (27.9%) were categorized as having severe disease; the median (IQR) age of the enrolled patients was 60.0 (49.0–68.0) years. The most common comorbidity was hypertension (712 patients [29.3%]). The median (IQR) duration of hospital stay was 14.0 (9.0-20.0) days, and the median (IQR) time from discharge to follow-up was 364.0 (357.0–371.0) days. During hospitalization, 54 patients (2.2%) were admitted to the intensive care unit (ICU). In total, 1743 patients (71.6%) received oxygen therapy, among which 21 patients (0.9%) received mechanical ventilation. Patients in the severe group were older, had more male individuals, had higher frequencies of coexisting disorders, and had a longer hospital stay, as well as a higher percentage of ICU admission (Table 1).

**Table 1.** Characteristics of Enrolled Patients

Characteristic	Patients, No. (%)				*P* value	
	Enrolled patients (N = 2433)	Severe (n = 680)	Nonsevere (n = 1753)	Lost to follow-up (n = 1555)	Enrolled patients vs lost to follow-up	Severe vs nonsevere
Age, median (IQR), y^a^	60.0 (49.0-68.0)	66.0 (57.0-72.0)	57.0 (46.0-66.0)	64.0 (52.0-71.0)	<.001	<.001
Sex						
Male	1205 (49.5)	361 (53.1)	844 (48.1)	775 (49.8)	.91	.03
Female	1228 (50.5)	319 (46.9)	909 (51.9)	780 (50.2)		
Severe disease	680 (27.9)	NA	NA	460 (29.6)	.27	NA
Cigarette smoking						
Never	2239 (92.0)	628 (92.3)	1611 (92.0)	1441 (92.7)	.76	.92
Former	39 (1.6)	10 (1.5)	29 (1.7)	23 (1.5)		
Active	155 (6.4)	42 (6.2)	113 (6.4)	91 (5.9)		
Coexisting disorders						
0	1394 (57.3)	303 (44.6)	1091 (62.2)	767 (49.3)	<.001	<.001
1	609 (25.0)	192 (28.2)	417 (23.8)	442 (28.4)		
≥2	430 (17.7)	185 (27.2)	245 (14.0)	346 (22.3)		
Hypertension	712 (29.3)	276 (40.6)	436 (24.9)	528 (34.0)	.001	<.001
Diabetes	337 (13.9)	133 (19.6)	204 (11.6)	230 (14.8)	.39	<.001
Cardiovascular diseases	223 (9.2)	101 (14.9)	122 (7.0)	188 (12.1)	.003	<.001
Chronic liver diseases	122 (5.0)	31 (4.6)	91 (5.2)	90 (5.8)	.28	.52
Cerebrovascular diseases	82 (3.4)	40 (5.9)	42 (2.4)	93 (6.0)	<.001	<.001
Chronic kidney diseases	58 (2.4)	19 (2.8)	39 (2.2)	48 (3.1)	.17	.41
Tumor	44 (1.8)	17 (2.5)	27 (1.5)	43 (2.8)	.04	.11
Tracheitis	40 (1.6)	16 (2.4)	24 (1.4)	38 (2.4)	.08	.09
COPD	23 (0.9)	12 (1.8)	11 (0.6)	28 (1.8)	.02	.009
Time from discharge to follow-up, d^a^	364.0 (357.0-371.0)	361.0 (356.0-369.0)	366.0 (358.0-372.0)	NA	NA	<.001
Length of hospital stay, d^a^	14.0 (9.0-20.0)	15.0 (10.0-24.0)	14.0 (8.5-20.0)	13.0 (9.0-20.0)	.17	<.001
ICU admission	54 (2.2)	43 (6.3)	11 (0.6)	54 (3.5)	.02	<.001
Oxygen therapy	1743 (71.6)	596 (87.6)	1147 (65.4)	1157 (74.4)	.06	<.001
Mechanical ventilation	21 (0.9)	21 (3.1)	0	26 (1.7)	.02	NA

Abbreviations: COPD, chronic obstructive pulmonary disease; ICU, intensive care unit; NA, not applicable.

^a^ Mann-Whitney *U* test. The rest of the statistical tests were calculated with the Pearson χ^2^ test.

Compared with enrolled patients, those who were lost to follow-up (N = 1555) were older, had a higher percentage of co-existing hypertension, cardiovascular diseases, cerebrovascular diseases and COPD, and had a higher percentage of ICU admission and mechanical ventilation. No significant difference was found in sex, disease severity, or the percentage of smokers (Table 1).

### Characteristics of Long-term Sequelae at 1-Year Follow-up

During hospitalization, 2322 out of 2433 patients (95.4%) reported at least 1 symptom. At 1-year follow-up, 1338 patients (55.0%) were completely free of any COVID-19–related symptom, whereas 1095 patients (45.0%) reported at least 1 symptom, and the percentage was higher in the patients who were in the severe group while hospitalized (severe vs nonsevere: 54.0% vs 41.5%; OR, 1.31 [95% CI, 1.04-1.65]; *P* = .02). Female patients had a higher percentage of any symptoms than male patients (female vs male: 47.9% vs 42.1%; OR, 0.81 [95% CI, 0.65-1.00]; *P* = .049). Of those with persisting symptoms, 412 (16.9%) reported 1 symptom, 299 (12.3%) reported 2 symptoms, and 384 (15.8%) reported 3 or more symptoms.

During hospitalization, the most common symptoms were fever (77.2% [1878 patients]), cough (70.0% [1703 patients]), fatigue (55.3% [1345 patients]), anorexia (50.6% [1232 patients]), and shortness of breath (41.1% [1000 patients]) (eTable 2 in the Supplement). At 1-year follow-up, no patient reported fever and the most common symptoms were fatigue (27.7% [696 patients]), sweating (16.9% [424 patients]), chest tightness (13.0% [326 patients]), anxiety (10.4% [262 patients]), and myalgia (7.9% [198 patients]) (Table 2). The percentage of symptoms during the acute phase of the disease and at 1-year follow-up is compared in the eFigure in the Supplement. The percentage of patients reporting cough decreased to 4.1% [104 patients], anorexia to 0.8% [20 patients], and shortness of breath to 4.1% [103 patients]. Besides sweating and anxiety, there were several other newly reported symptoms, including palpitation (4.2% [106 patients]), edema of lower limbs (1.4% [36 patients]), taste change (1.4% [35 patients]), and impaired sense of smell (1.3% [32 patients]). The prevalence of several symptoms in the severe group was significantly higher than those in the nonsevere group (severe vs nonsevere fatigue: 35.9% vs 25.8%; OR, 1.36 [95% CI, 1.10-1.68]; *P* = .004; severe vs nonsevere chest tightness: 20.4% vs 10.7%; OR, 1.68 [95% CI, 1.29-2.19]; *P* < .001; severe vs nonsevere shortness of breath: 6.6% vs 3.3%; OR, 1.84 [95% CI, 1.17-2.88]; *P* = .008; severe vs nonsevere impaired sense of smell: 2.5% vs 0.9%; OR, 2.59 [95% CI, 1.19-5.65]; *P* = .02; severe vs nonsevere sore throat: 1.8% vs 0.7%; OR, 3.10 [95% CI, 1.31-7.32]; *P* = .01). Female patients had significantly higher percentages of anxiety (13.4% vs 8.1%, *P* = .001), myalgia (9.6% vs 6.6%, *P* = .01), and headache (3.1% vs 1.6%, *P* = .001) (eTable 3 in the Supplement).

**Table 2.** Symptoms at 1-Year Follow-up According to Disease Severity

Symptoms	Patients, No. (%)			Severe vs nonsevere	
	Enrolled patients (n = 2433)	Severe (n = 680)	Nonsevere (n = 1753)	OR (95% CI)	*P* value
Any one of the following symptoms	1095 (45.0)	367 (54.0)	728 (41.5)	1.31 (1.04-1.65)	.02
Fatigue	696 (27.7)	244 (35.9)	452 (25.8)	1.36 (1.10-1.68)	.004
Sweating	424 (16.9)	156 (22.9)	268 (15.3)	1.26 (0.99-1.61)	.06
Chest tightness	326 (13.0)	139 (20.4)	187 (10.7)	1.68 (1.29-2.19)	<.001
Anxiety	262 (10.4)	82 (12.1)	180 (10.3)	0.99 (0.72-1.34)	.92
Myalgia	198 (7.9)	76 (11.2)	122 (7.0)	1.36 (0.97-1.90)	.08
Palpitation	106 (4.2)	40 (5.9)	66 (3.8)	1.07 (0.68-1.68)	.78
Cough	104 (4.1)	46 (6.8)	58 (3.3)	1.55 (1.00-2.41)	.05
Shortness of breath	103 (4.1)	45 (6.6)	58 (3.3)	1.84 (1.17-2.88)	.008
Dizziness	82 (3.3)	26 (3.8)	56 (3.2)	0.92 (0.55-1.53)	.75
Expectoration	75 (3.0)	34 (5.0)	41 (2.3)	1.44 (0.86-2.43)	.17
Dyspnea	69 (2.7)	30 (4.4)	39 (2.2)	1.25 (0.73-2.15)	.41
Headache	57 (2.3)	22 (3.2)	35 (2.0)	1.41 (0.78-2.57)	.26
Edema of lower limbs	36 (1.4)	19 (2.8)	17 (1.0)	1.76 (0.82-3.75)	.15
Taste change	35 (1.4)	15 (2.2)	20 (1.1)	1.33 (0.64-2.77)	.45
Impaired sense of smell	32 (1.3)	17 (2.5)	15 (0.9)	2.59 (1.19-5.65)	.02
Sore throat	25 (1.0)	12 (1.8)	13 (0.7)	3.10 (1.31-7.32)	.01
Anorexia	20 (0.8)	8 (1.2)	12 (0.7)	1.60 (0.39-2.88)	.91
Diarrhea	18 (0.7)	6 (0.9)	12 (0.7)	1.62 (0.57-4.56)	.36
Hemoptysis	5 (0.2)	0	5 (0.3)	NA	NA
Nausea	5 (0.2)	1 (0.1)	4 (0.2)	0.39 (0.04-3.66)	.41
Chill	3 (0.1)	0	3 (0.2)	NA	NA
Vomiting	3 (0.1)	0	3 (0.2)	NA	NA
Fever	0	0	0	NA	NA

After propensity score–matching, 1453 patients (59.7%) in the enrolled population were matched successfully with those lost to follow-up. As shown in eTable 4 in the Supplement, the baseline characteristics were comparable. In the propensity score–matched population, the most common symptoms were fatigue (29.2% [425 patients]), sweating (16.7% [243 patients]), chest tightness (13.4% [195 patients]), anxiety (10.7% [155 patients]), and myalgia (8.5% [124 patients]), which were similar to that of the overall enrolled population (eTable 5 in the Supplement).

### Risk Factors of Post-Infection Sequelae at 1-Year Follow-up

We next investigated risk factors for fatigue, which was the most common symptom at 1-year follow-up (eTable 6 in the Supplement). On univariate analysis, age, sex, disease severity, oxygen therapy, mechanical ventilation, length of hospital stay, and follow-up times were associated with fatigue. After multivariable adjustment, severe disease (OR, 1.43; 95% CI, 1.18-1.74; *P* < .001), older age (OR, 1.02; 95% CI, 1.01-1.02; *P* < .001), and female sex (OR, 1.27; 95% CI, 1.06-1.52; *P* = .008) were associated with a higher risk of fatigue. For dyspnea, age, oxygen therapy, mechanical ventilation, and coexisting chronic liver diseases were risk factors after multivariable adjustment (eTable 7 in the Supplement).

In patients with at least 3 symptoms at 1-year of follow-up, older age (OR, 1.02; 95% CI, 1.01-1.03; *P* < .001) and severe disease (OR, 1.51; 95% CI, 1.14-1.99; *P* = .004) were independent risk factors (eTable 8 in the Supplement).

### CAT Scoring at 1-Year Follow-up

**Figure 2.**
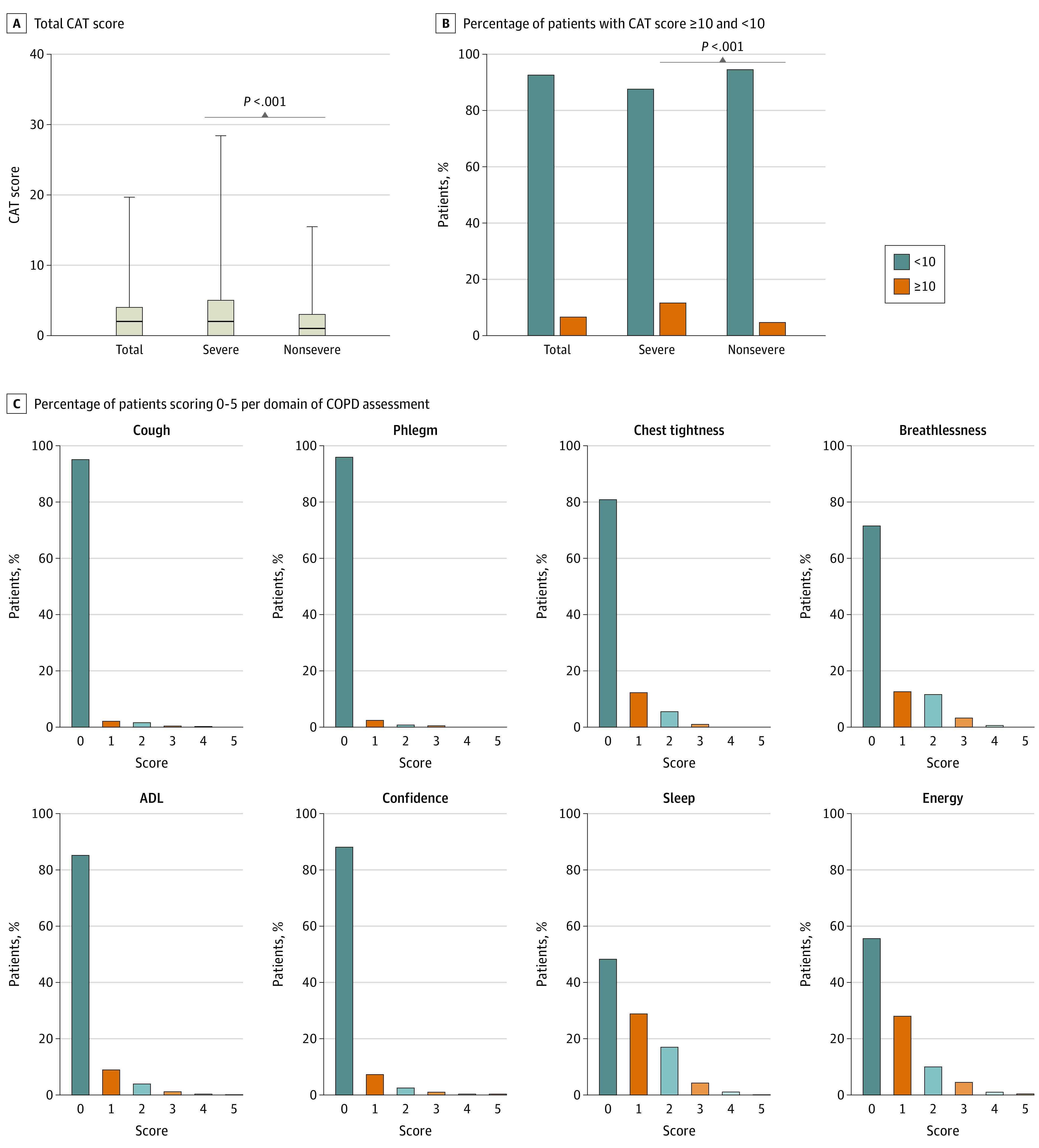
Total and Item CAT Scores in Patients at 1-Year Follow-up ADL indicates activities of daily living; CAT, COPD assessment test; COPD, chronic obstructive pulmonary disease.

Previously, CAT was shown to be a useful tool to assess symptom burden of patients with COVID-19.^14^ In the present study, all 2433 patients completed CAT assessment. The median (IQR) CAT score was 2 (0–4) in the total cohort, and the severe group had a significantly higher median (IQR) CAT score ( 2 [0–5]) compared with the nonsevere group (1 [0–3]; *P* < .001) (Figure 2A). A total of 161 patients (6.6%) had CAT total scores of at least 10. The severe group had a significantly higher proportion of patients with a CAT score of at least 10 compared with the nonsevere group (severe vs nonsevere: 79 [11.6%] vs 82 [4.7%]; χ^2^=38.187; *P* < .001) (Figure 2B). The CAT item scores are shown in Figure 2C.

We next assessed risk factors associated with CAT scores of at least 10. On univariate analysis, severe disease (OR, 2.68; 95% CI, 1.94-3.71; *P* < .001), ICU admission (OR, 2.93; 95% CI, 1.40-6.10; *P* = .004), mechanical ventilation (OR, 3.59; 95% CI, 1.19-10.85; *P* = .02), hypertension (OR, 1.66; 95% CI, 1.20-2.31; *P* = .002), cardiovascular diseases (OR, 2.36; 95% CI, 1.54-3.62; *P* < .001), and cerebrovascular diseases (OR, 3.80; 95% CI, 2.14-6.74; *P* < .001) were associated with higher odds of CAT scores of at least 10. After multivariable adjustment, severe disease (OR, 1.84; 95% CI, 1.31-2.58; *P* < .001), and coexisting cerebrovascular diseases (OR, 1.95; 95% CI, 1.07-3.54; *P* = .03) were found to be independent risk factors for CAT scores of at least 10 (Table 3).

**Table 3.** Logistic Regression Models to Evaluate the Risk Factors for CAT Scores ≥10

Variables^a^^,^^b^	Univariable, OR (95% CI)	*P* value	Multivariable, OR (95% CI)	*P* value
Age, per year	1.07 (1.05-1.08)	<.001	1.06 (1.04-1.07)	<.001
Sex, vs female	1.07 (0.78-1.48)	.66	NA	NA
Severity, vs no	2.68 (1.94-3.71)	<.001	1.84 (1.31-2.58)	<.001
ICU admission, vs no	2.93 (1.40-6.10)	.004	NA	NA
Oxygen therapy, vs no	1.00 (0.70-1.42)	>.99	NA	NA
Mechanical ventilation, vs no	3.59 (1.19-10.85)	.02	NA	NA
Cigarette smoking, vs never				
Former	0.31 (0.04-2.28)	.25	NA	NA
Active	1.08 (0.59-1.96)	.81	NA	NA
Length of hospital stay, per day	1.00 (0.98-1.02)	.79	NA	NA
Follow-up time, per day	0.98 (0.97-1.00)	.04	NA	NA
Coexisting disorder, vs no				
Hypertension	1.66 (1.20-2.31)	.002	NA	NA
Diabetes	1.28 (0.83-1.97)	.27	NA	NA
Cardiovascular diseases	2.36 (1.54-3.62)	<.001	NA	NA
Chronic liver diseases	1.28 (0.65-2.49)	.48	NA	NA
Cerebrovascular diseases	3.80 (2.14-6.74)	<.001	1.95 (1.07-3.54)	.03
Chronic kidney diseases	0.76 (0.24-2.47)	.65	NA	NA
Tumor	0.67 (0.16-2.78)	.58	NA	NA
COPD	2.13 (0.63-7.26)	.23	NA	NA

Abbreviations: CAT, COPD Assessment Test; COPD, chronic obstructive pulmonary disease; ICU, intensive care unit; NA, not applicable.

^a^ Dependent variable: CAT≥10 vs CAT<10.

^b^ Independent variables: age, severity, ICU admission, oxygen therapy (excluding mechanical ventilation), mechanical ventilation, follow-up time, hypertension, cardiovascular diseases, and cerebrovascular diseases.

## Discussion

This study reports the long-term health outcomes of COVID-19 survivors at 1-year after hospital discharge in a large cohort of patients. We found that 1095 patients (45.0%) reported at least 1 symptom, and the most common symptoms were fatigue, sweating, chest tightness, anxiety, and myalgia. Older age, female gender, and severe disease were associated with higher risks of fatigue or more symptoms. In total, 161 patients (6.6%) had CAT total scores of at least 10, for which the risk factors included severe disease during hospital stay or coexisting cerebrovascular diseases. Taken together, our study found that patients with severe disease had increased risks of more symptoms and higher CAT scores at 1-year follow-up.

COVID-19 can affect multiple organs, which leads to both acute organ damage and long-term sequelae, with the latter effects gaining increasing concerns.^20^ We found that fatigue was the most commonly reported symptom, which is consistent with previous studies.^10,12,21^ Fatigue is common after acute lung injury and is associated with substantial impairments in physical function and quality of life.^22^ High percentage of fatigue was possibly due to multiple organ injury following infection of SARS-CoV-2. Fatigue occurred in 27.7% of individuals in this study, which is lower than the 53% to 71% as reported by other studies.^5,10,23,24^ This is likely to be due to longer follow-up time, allowing the patients to gradually recover from previously existing symptoms.

Compared with male individuals, female patients had a significantly higher percentage of anxiety, myalgia, and headache. In a 3-month follow-up survey of patients with COVID-19, women were found to have higher percentages of fatigue, postactivity polypnea, and alopecia.^8^ Similarly, higher levels of stress, depression, and anxiety were also found in female SARS survivors.^25^ These studies all suggest that women are at greater risk of long-term symptomatic sequelae. Because anxiety was one of the most common symptoms at 1-year follow-up, the need for mental health assessment in COVID-19 survivors may be considered to help identify patients requiring psychological intervention.

In the present study, 4.2% of patients reported palpitations, which may point to long-term damage of COVID-19 to the cardiovascular system. Previously, cardiac involvement was found in 78% of German patients recovered from COVID-19 and ongoing myocardial inflammation in 60% of patients,^26^ but these cases were reported shortly after the acute illness. Also, cardiac injury was associated with an increased risk of in-hospital mortality.^26,27,28,29^ Because COVID-19 is still rapidly spreading over the world, more attention should be paid to cardiac damage in both the acute and postinfection phase of the disease. Changes in taste and smell were reported by a considerable proportion of patients, and these were also frequently reported in the acute phase of the disease,^30,31^ which suggests that COVID-19 may impact multiple sensory modalities and result in disruption of chemosensory function. In a series of mild-to-moderate symptomatic patients with COVID-19, 21.3% of patients reported persistent altered sense of smell or taste 1 year after disease onset.^32^ Taken together, our research suggests that the health consequences of COVID-19 extend far beyond acute infection.

Currently there’s no consensus on how to quantify the burden of COVID-19 symptoms. The 8-point differential item response scale of CAT—including cough, sputum, chest tightness, breathlessness, activity limitation, confidence leaving the home, sleep, and energy—had a significant overlap with the commonly reported COVID-19–related symptoms. The CAT conducted via telephone interview had a high validity and was comparable to face-to-face interviews,^33^ so CAT scoring may be a simple and useful tool to assess symptom burden of patients with COVID-19. In the present study, a total of 6.6% of patients had CAT total scores of at least 10, which was much lower than that reported by a previous study conducted at shorter follow-up time.^14^ In a study of 481 people in Canada without COPD, the mean (SD) CAT score was 6.9 (6.2).^34^ This indicates that the symptom burden decreased dramatically at 1-year follow-up and few people have substantial respiratory health status impairment, as measured by the CAT. In such individuals, we have found that severe disease during the acute illness and coexisting cerebrovascular diseases are risk factors. This study was done at 1 year, so further studies with longer follow-up time are still needed.

### Limitations

There are several limitations in this study. First, as much as 19% of the eligible population was not accessible and nearly 25% of the remaining population declined to participate the current study, and some of the demographic characteristics differed between enrolled patients and those lost to follow-up, predisposing a risk of survivor bias and the included patients may be less representative of the target population. However, the propensity matching that we performed in the sensitivity analysis suggests that this bias may be small. Second, telephone interviews might not be as accurate as face-to-face interviews, and the CAT scores of patients during hospitalization were not obtained so we do not know the state of the patients at discharge. Third, case definitions for COVID-19 remain poorly defined, and there was no comparison group (control group). Multiple variants of the virus that causes COVID-19 have been documented globally and these may have different virulence and long-term sequelae, which may limit the generalizability of our findings to infections with later variants.

## Conclusions

This study found that among patients who had been hospitalized with COVID-19 in Wuhan at the beginning of the pandemic, the most common symptoms at 1-year after discharge were fatigue, sweating, chest tightness, anxiety, and myalgia. Patients with severe disease had more postinfection symptoms and higher CAT scores. The findings provide valuable information about the long-term health outcomes of COVID-19 survivors and identify risk factors for sustained symptoms and poor respiratory health status, which is of importance with the coming of the post-COVID-19 era.

## Notes

Supplement. **eFigure 1.** Percentage of Patients Presenting With COVID-19-Related Symptoms During The Acute Phase of The Disease (Left) And at 1-year Follow-Up (Right) **eTable 1.** COVID-19 Survivors Clinical Sequelae Follow-up Questionnaire **eTable 2.** Symptoms of Enrolled Patients And Those Lost to Follow-Up During COVID-19 Hospitalization **eTable 3.** Symptoms at One-Year Follow-Up According to Sex **eTable 4.** Characteristics of Enrolled Patients After Propensity Score Matching **eTable 5.** Symptoms at One-Year Follow-Up in Overall And Propensity Score-Matched Population **eTable 6.** Logistic Regression Models to Evaluate The Risk Factors For Fatigue **eTable 7.** Logistic Regression Models to Evaluate The Risk Factors For Dyspnea **eTable 8.** Logistic Regression Models to Evaluate The Risk Factors For Symptom Numbers ≥ 3
