# Supplementary material for: Symptoms and Health Outcomes Among Survivors of COVID-19 Infection 1 Year After Discharge From Hospitals in Wuhan, China
Source: JAMA Netw Open. 2021 Sep 29;4(9):e2127403. doi: 10.1001/jamanetworkopen.2021.27403 (PMC8482055; doi:10.1001/jamanetworkopen.2021.27403)
Supplement: Supplement. — eFigure 1. Percentage of Patients Presenting With COVID-19-Related Symptoms During The Acute Phase of The Disease (Left) And at 1-year Follow-Up (Right) eTable 1. COVID-19 Survivors Clinical Sequelae Follow-up Questionnaire eTable 2. Symptoms of Enrolled Patients And Those Lost to Follow-Up During COVID-19 Hospitalization eTable 3. Symptoms at One-Year Follow-Up According to Sex eTable 4. Characteristics of Enrolled Patients After Propensity Score Matching eTable 5. Symptoms at One-Year Follow-Up in Overall And Propensity Score-Matched Population eTable 6. Logistic Regression Models to Evaluate The Risk Factors For Fatigue eTable 7. Logistic Regression Models to Evaluate The Risk Factors For Dyspnea eTable 8. Logistic Regression Models to Evaluate The Risk Factors For Symptom Numbers ≥ 3 [file jamanetwopen-e2127403-s001.pdf]

Supplemental Online Content

Zhang X, Wang F, Shen Y, et al. Symptoms and health outcomes among survivors of COVID-19 infection 1 year after discharge from hospitals in Wuhan, China. *JAMA Netw Open.* 2021;4(9):e2127403. doi:10.1001/jamanetworkopen.2021.27403

eFigure 1. Percentage of Patients Presenting With COVID-19-Related Symptoms During The Acute Phase of The Disease (Left) And at 1-year Follow-Up (Right)

eTable 1. COVID-19 Survivors Clinical Sequelae Follow-up Questionnaire

eTable 2. Symptoms of Enrolled Patients And Those Lost to Follow-Up During COVID-19 Hospitalization

eTable 3. Symptoms at One-Year Follow-Up According to Sex

eTable 4. Characteristics of Enrolled Patients After Propensity Score Matching

eTable 5. Symptoms at One-Year Follow-Up in Overall And Propensity Score-Matched Population

eTable 6. Logistic Regression Models to Evaluate The Risk Factors For Fatigue

eTable 7. Logistic Regression Models to Evaluate The Risk Factors For Dyspnea

eTable 8. Logistic Regression Models to Evaluate The Risk Factors For Symptom Numbers ≥ 3

This supplemental material has been provided by the authors to give readers additional information about their work.

© 2021 Zhang X et al. *JAMA Network Open.*

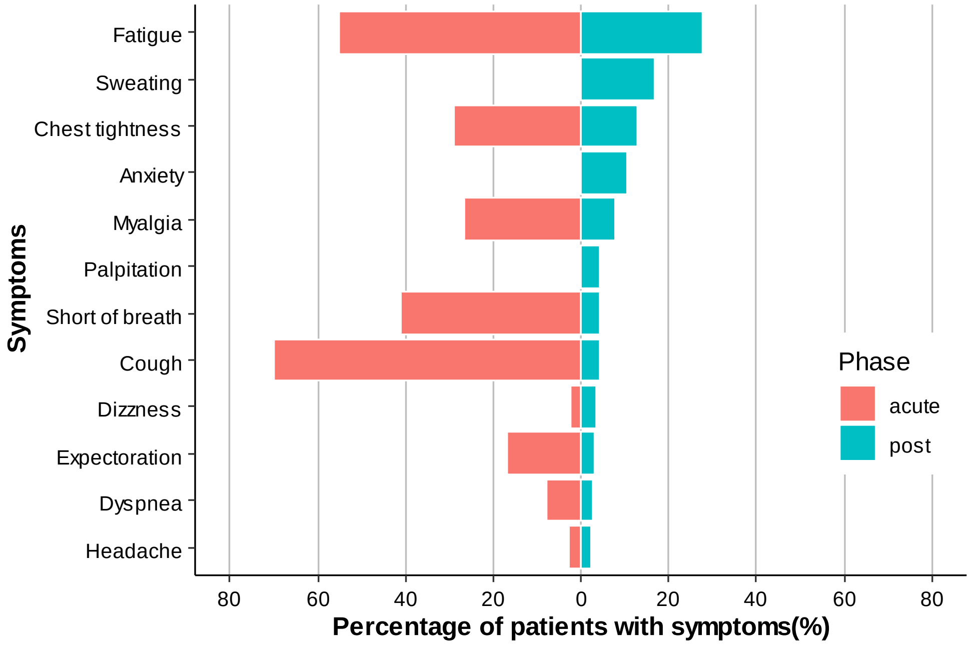

eFigure 1. Percentage of patients presenting with COVID-19-related symptoms during the acute phase of the disease (left) and at 1-year follow-up (right).

© 2021 Zhang X et al. *JAMA Network Open.*

eTable 1. COVID-19 Survivors Clinical Sequelae Follow-up Questionnaire.

Number: ____ Name: ____ Willingness to follow up: ☐yes ☐no ☐lost-to-follow up

1. Follow-up of COVID-19-related symptoms

Are the following symptoms currently present?

1. Fever: ☐yes ☐no	2. Cough: ☐yes ☐no
3. Fatigue: ☐yes ☐no	4. Anorexia: ☐yes ☐no
5. Short of breath: ☐yes ☐no	6. Myalgia: ☐yes ☐no
7. Chest tightness: ☐yes ☐no	8. Expectoration: ☐yes ☐no
9. Dyspnea: ☐yes ☐no	10. Diarrhea: ☐yes ☐no
11. Sore throat: ☐yes ☐no	12. Nausea: ☐yes ☐no
13. Dizziness: ☐yes ☐no	14. Headache: ☐yes ☐no
15. Vomiting: ☐yes ☐no	16. Chill: ☐yes ☐no
17. Hemoptysis: ☐yes ☐no	
Other COVID-19-related symptoms, if any:	

2.CAT scoring Total score: ________

Symptoms	score						Symptoms
I never cough	0	1	2	3	4	5	I cough all the time
I have no phlegm (mucus) in my chest at all	0	1	2	3	4	5	My chest is completely full of phlegm (mucus)
My chest does not feel tight at all	0	1	2	3	4	5	My chest feels very tight
When I walk up a hill or one flight of stairs I am not breathless	0	1	2	3	4	5	When I walk up a hill or one flight of stairs I am very breathless
I am not limited doing any activities at home	0	1	2	3	4	5	I am very limited doing activities at home
I am confident leaving my home despite my lung condition	0	1	2	3	4	5	I am not at all confident leaving my home because of my lung condition
I sleep soundly	0	1	2	3	4	5	I don’t sleep soundly because of my lung condition
I have lots of energy	0	1	2	3	4	5	I have no energy at all

© 2021 Zhang X et al. *JAMA Network Open.*

eTable 2. Symptoms of enrolled patients and those lost to follow-up during COVID-19 hospitalization.

	Enrolled patients (n=2433)	Severe (n=680)	Non-severe (n=1753)	Lost to follow-up (n=1555)	P value enrolled patients vs. Lost to follow-up	P value Severe vs. Non-Severe
Symptoms						
Anyone of the following symptoms	2322(95.4)	655(96.3)	1667(95.1)	1467(94.3)	0.22	0.19
Fever	1878(77.2)	558(82.1)	1320(75.3)	1216(78.2)	0.46	<0.001
Cough	1703(70.0)	502(73.8)	1201(68.5)	1063(68.4)	0.33	0.01
Fatigue	1345(55.3)	399(58.7)	946(54.0)	835(53.7)	0.37	0.04
Anorexia	1232(50.6)	361(53.1)	871(49.7)	859(55.2)	0.003	0.13
Short of breath	1000(41.1)	356(52.4)	644(36.7)	628(40.4)	0.70	<0.001
Chest tightness	703(28.9)	243(35.7)	460(26.2)	445(28.6)	0.89	<0.001
Myalgia	650(26.7)	180(26.5)	470(26.8)	422(27.1)	0.73	0.87
Expectoration	413(17.0)	138(20.3)	275(15.7)	300(19.3)	0.06	0.007
Dyspnea	188(7.7)	109(16.0)	79(4.5)	101(6.5)	0.15	<0.001
Diarrhea	165(6.8)	42(6.2)	123(7.0)	100(6.4)	0.68	0.46
Sore throat	148(6.1)	32(4.7)	116(6.6)	74(4.8)	0.08	0.08
Nausea	70(2.9)	19(2.8)	51(2.9)	46(3.0)	0.87	0.88
Vomiting	66(2.7)	20(2.9)	46(2.6)	35(2.3)	0.37	0.67
Headache	64(2.6)	11(1.6)	53(3.0)	40(2.6)	0.92	0.05
Chill	58(2.4)	30(4.4)	28(1.6)	36(2.3)	0.90	<0.001
Dizziness	57(2.3)	16(2.4)	41(2.3)	48(3.1)	0.15	0.98

© 2021 Zhang X et al. *JAMA Network Open.*

Hemoptysis	16(0.7)	6(0.9)	10(0.6)	9(0.6)	0.76	0.57

eTable 3. Symptoms at one-year follow-up according to sex.

	Patients (n=2433)	Female (n=1228)	Male (n=1205)	OR(95%CI) Male vs. Female	P value Male vs. Female
Symptoms					
Any one of the following symptoms	1095(45.0)	588(47.9)	507(42.1)	0.81(0.65-1.00)	0.05
Symptom numbers					
0	1338(55.0)	640(52.1)	698(57.9)		
1	412(16.9)	214(17.4)	198(16.4)		
2	299(12.3)	162(13.2)	137(11.4)		
≥3	384(15.8)	212(17.3)	172(14.3)		
Fatigue	696(27.7)	381(31.0)	315(26.1)	0.79(0.63-1.00)	0.05
Sweating	424(16.9)	233(19.0)	191(15.9)	0.88(0.67-1.17)	0.39
Chest tightness	326(13.0)	170(13.8)	156(12.9)	0.94(0.68-1.28)	0.68
Anxiety	262(10.4)	164(13.4)	98(8.1)	0.56(0.40-0.78)	0.001
Myalgia	198(7.9)	118(9.6)	80(6.6)	0.62(0.43-0.91)	0.01
Palpitation	106(4.2)	53(4.3)	53(4.4)	0.95(0.59-1.54)	0.85
Cough	104(4.1)	54(4.4)	50(4.1)	0.94(0.59-1.50)	0.79
Short of breath	103(4.1)	49(4.0)	54(4.5)	1.24(0.75-2.06)	0.40
Dizzness	82(3.3)	48(3.9)	34(2.8)	0.67(0.39-1.16)	0.15
Expectoration	75(3.0)	31(2.5)	44(3.7)	1.46(0.83-2.56)	0.19
Dyspnea	69(2.7)	28(2.3)	41(3.4)	1.08(0.56-2.08)	0.82

© 2021 Zhang X et al. *JAMA Network Open.*

Headache	57(2.3)	38(3.1)	19(1.6)	0.30(0.14-0.62)	0.001
Edema of lower limbs	36(1.4)	17(1.4)	19(1.6)	1.27(0.59-2.75)	0.55
Taste change	35(1.4)	18(1.5)	17(1.4)	0.78(0.34-1.78)	0.56
Impaired sense of smell	32(1.3)	17(1.4)	15(1.2)	0.95(0.43-2.12)	0.91
Sore throat	25(1.0)	14(1.1)	11(0.9)	0.62(0.22-1.75)	0.37
Anorexia	20(0.8)	10(0.8)	10(0.8)	0.73(0.23-2.26)	0.58
Diarrhea	18(0.7)	11(0.9)	7(0.6)	0.83(0.29-2.41)	0.74
Hemoptysis	5(0.2)	2(0.2)	3(0.2)	0.64(0.06-6.66)	0.71
Nausea	5(0.2)	1(0.1)	4(0.3)	2.18(0.20-24.22)	0.53
Rhinobyon	4(0.2)	2(0.2)	2(0.2)	0.11(0.00-4.91)	0.26
Chill	3(0.1)	0	3(0.2)	NA	
Vomiting	3(0.1)	1(0.1)	1(0.1)	8.96(0.20-393.74)	0.26
Fever	0	0	0	NA	

© 2021 Zhang X et al. *JAMA Network Open.*

eTable 4. Characteristics of enrolled patients after propensity score matching.

	Matched Follow-up	Matched Lost to follow-up	P value
Numbers	1453	1453	
Age – Median (IQR), years[a]	62.0(51.0-70.0)	63.0(52.0-70.0)	0.69
Sex – No. (%)			0.35
Male	699(48.1)	724(49.8)	
Female	754(51.9)	729(50.2)	
Severe disease – No. (%)	406(27.9)	409(28.1)	0.90
Cigarette smoking – No. (%)			0.37
Never	1333(91.7)	1353(93.1)	
Former	24(1.7)	21(1.5)	
Active	96(6.6)	79(5.4)	
Coexisting disorders – No. (%)			
Coexisting disorders number			0.51
0	771(53.1)	752(51.8)	
1	386(26.6)	414(28.5)	
≥2	296(20.3)	287(19.7)	
Hypertension	478(32.9)	464(31.9)	0.58
Diabetes mellitus	214(14.7)	205(14.1)	0.64
Cardiovascular diseases	146(10.0)	156(10.7)	0.54
Chronic liver diseases	73(5.0)	82(5.6)	0.46
Cerebrovascular diseases	61(4.2)	58(4.0)	0.78
Chronic kidney diseases	34(2.3)	39(2.7)	0.55

© 2021 Zhang X et al. *JAMA Network Open.*

Tumour	24(1.7)	37(2.5)	0.09
Tracheitis	26(1.8)	31(2.1)	0.50
COPD	15(1.0)	19(1.3)	0.49
Length of hospital stay, days [a]	14.0(9.0-21.0)	14.0(9.0-20.0)	0.06
ICU admission – No. (%)	39(2.7)	31(2.1)	0.33
Oxygen therapy, No. (%)	1050(72.3)	1059(72.9)	0.71
Mechanical Ventilation, No. (%)	13(0.9)	6(0.4)	0.12

Abbreviation: IQR, Inter-quartile range; ICU, Intensive Care Unit; COPD, Chronic obstructive pulmonary disease; NA, not applicable.

a, Mann-Whitney U test. The rest: Pearson χ^2 test.

© 2021 Zhang X et al. *JAMA Network Open.*

eTable 5. Symptoms at one-year follow-up in Overall and Propensity Score-Matched population.

Symptoms	Overall population			Propensity Score-Matched population			P value (Overall vs Propensity Score-Matched population)		
	**Total**	**Severe**	**Non-severe**	**Total**	**Severe**	**Non-severe**	**Total**	**Severe**	**Non-severe**
Number	2433	680	1753	1453	406	1047			
Any one of the following symptoms	1095(45.0)	367(54.0)	728(41.5)	654(45.0)	211(52.0)	443(42.3)	1.00	0.52	0.69
Fatigue	696(27.7)	244(35.9)	452(25.8)	425(29.2)	135(33.3)	290(27.7)	0.67	0.38	0.27
Sweating	424(16.9)	156(22.9)	268(15.3)	243(16.7)	85(20.9)	158(15.1)	0.57	0.44	0.89
Chest tightness	326(13.0)	139(20.4)	187(10.7)	195(13.4)	73(18.0)	122(11.7)	0.99	0.32	0.42
Anxiety	262(10.4)	82(12.1)	180(10.3)	155(10.7)	48(11.8)	107(10.2)	0.92	0.91	0.97
Myalgia	198(7.9)	76(11.2)	122(7.0)	124(8.5)	41(10.1)	83(7.9)	0.67	0.58	0.34
Palpitation	106(4.2)	40(5.9)	66(3.8)	60(4.1)	22(5.4)	38(3.6)	0.74	0.75	0.85
Cough	104(4.1)	46(6.8)	58(3.3)	68(4.7)	30(7.4)	38(3.6)	0.55	0.70	0.65
Short of breath	103(4.1)	45(6.6)	58(3.3)	62(4.3)	25(6.2)	37(3.5)	0.96	0.77	0.75
Dizzness	82(3.3)	26(3.8)	56(3.2)	49(3.4)	14(3.4)	35(3.3)	1.00	0.75	0.83
Expectoration	75(3.0)	34(5.0)	41(2.3)	56(3.9)	24(5.9)	32(3.1)	0.20	0.52	0.25
Dyspnea	69(2.7)	30(4.4)	39(2.2)	41(2.8)	14(3.4)	27(2.6)	0.98	0.44	0.55
Headache	57(2.3)	22(3.2)	35(2.0)	28(1.9)	12(3.0)	16(1.5)	0.39	0.80	0.37
Edema of lower limbs	36(1.4)	19(2.8)	17(1.0)	22(1.5)	11(2.7)	11(1.1)	0.93	0.93	0.84
Taste change	35(1.4)	15(2.2)	20(1.1)	20(1.4)	9(2.2)	11(1.1)	0.87	0.99	0.83
Impaired sense of	32(1.3)	17(2.5)	15(0.9)	14(1.0)	10(2.5)	4(0.4)	0.33	0.97	0.14

© 2021 Zhang X et al. *JAMA Network Open.*

smell									
Sore throat	25(1.0)	12(1.8)	13(0.7)	15(1.0)	6(1.5)	9(0.9)	0.99	0.72	0.73
Anorexia	20(0.8)	8(1.2)	12(0.7)	10(0.7)	4(1.0)	6(0.6)	0.65	1.00[a]	0.72
Diarrhea	18(0.7)	6(0.9)	12(0.7)	12(0.8)	3(0.7)	9(0.9)	0.77	1.00[a]	0.60
Hemoptysis	5(0.2)	0	5(0.3)	5(0.3)	0	5(0.5)	0.52[a]	1.00[a]	0.52[a]
Nausea	5(0.2)	1(0.1)	4(0.2)	2(0.1)	0	2(0.2)	1.00[a]	1.00[a]	1.00[a]
Rhinobyon	4(0.2)	1(0.1)	3(0.2)	4(0.3)	1(0.2)	3(0.3)	0.48[a]	1.00[a]	0.68[a]
Chill	3(0.1)	0	3(0.2)	3(0.2)	0	3(0.3)	0.68[a]	1.00[a]	0.68[a]
Vomiting	3(0.1)	0	3(0.2)	3(0.2)	0	3(0.3)	0.68[a]	1.00[a]	0.68[a]
Fever	0	0	0	0	0	0	1.00[a]	1.00[a]	1.00[a]

a, Fisher's exact test

© 2021 Zhang X et al. *JAMA Network Open.*

eTable 6. Logistic regression models to evaluate the risk factors for fatigue.

Variables	Univariable ORs(95%CI)	*P* value	Multivariable ORs(95%CI)	*P* value
Age, per year	1.02(1.01-1.03)	<0.001	1.02(1.01-1.02)	<0.001
Sex, vs. female	1.27(1.07-1.51)	0.008	1.27(1.06-1.52)	0.008
Severity, vs. no	1.61(1.33-1.95)	<0.001	1.43(1.18-1.74)	<0.001
ICU admission, vs. no	1.48(0.85-2.59)	0.17		
Oxygen therapy, vs. no	1.30(1.06-1.58)	0.01		
Mechanical ventilation, vs. no	3.08(1.27-7.47)	0.01		
Cigarette smoking，vs. never				
Former	0.86(0.41-1.77)	0.67		
Active	0.78(0.53-1.14)	0.20		
Length of hospital stay, per day	1.01(1.00-1.02)	0.02		
Follow-up time, per day	0.97(0.95-0.99)	0.005		
Coexisting disorder, vs. no				
Hypertension	1.20(1.00-1.46)	0.06		
Diabetes	1.15(0.90-1.48)	0.27		
Cardiovascular diseases	1.01(0.74-1.36)	0.97		
Chronic liver diseases	1.14(0.77-1.69)	0.52		
Cerebrovascular diseases	1.10(0.68-1.77)	0.70		

© 2021 Zhang X et al. *JAMA Network Open.*

Chronic kidney diseases	0.72(0.38-1.34)	0.29		
Tumour	1.05(0.55-2.01)	0.89		
COPD	0.88(0.35-2.24)	0.79		

Dependent variable: fatigue at one-year follow-up.

Independent variables: age, sex, severity, Oxygen therapy (excluding mechanical ventilation), Mechanical ventilation, Length of hospital stay, Follow-up time, hypertension.

Abbreviations: ICU, intensive care unit; COPD, Chronic Obstructive Pulmonary Disease.

© 2021 Zhang X et al. *JAMA Network Open.*

eTable 7. Logistic regression models to evaluate the risk factors for dyspnea.

Variables	Univariable ORs(95%CI)	*P* value	Multivariable ORs(95%CI)	*P* value
Age, per year	1.04(1.02-1.06)	<0.001	1.03(1.01-1.05)	0.001
Sex, vs. female	0.66(0.41-1.08)	0.10		
Severity, vs. no	2.03(1.25-3.29)	0.004		
ICU admission, vs. no	2.06(0.63-6.78)	0.23		
Oxygen therapy, vs. no	1.99(1.06-3.73)	0.03	2.16(1.05-4.42)	0.04
Mechanical ventilation, vs. no	6.28(1.80-21.94)	0.004	9.94(2.40-41.15)	0.002
Cigarette smoking，vs. never				
Former	0.96(0.13-7.16)	0.97		
Active	1.72(0.76-3.91)	0.19		
Length of hospital stay, per day	1.03(1.01-1.05)	0.01		
Follow-up time, per day	0.97(0.95-0.99)	0.005		
Coexisting disorder, vs. no				
Hypertension	1.78(1.10-2.90)	0.02		
Diabetes	1.19(0.62-2.28)	0.61		
Cardiovascular diseases	1.71(0.86-3.40)	0.12		
Chronic liver diseases	3.85(1.97-7.54)	<0.001	4.17(2.10-8.25)	<0.001
Cerebrovascular diseases	1.32(0.41-4.27)	0.65		

© 2021 Zhang X et al. *JAMA Network Open.*

Chronic kidney diseases	1.23(0.29-5.15)	0.78		
Tumour	1.65(0.39-6.96)	0.50		
COPD	5.33(1.55-18.37)	0.008		

Dependent variable: dyspnea at one-year follow-up.

Independent variables: age, sex, severity, Oxygen therapy (excluding mechanical ventilation), Mechanical ventilation, Length of hospital stay, Follow-up time, hypertension, Chronic liver diseases, COPD.

Abbreviations: ICU, intensive care unit; COPD, Chronic Obstructive Pulmonary Disease.

© 2021 Zhang X et al. *JAMA Network Open.*

eTable 8. Logistic regression models to evaluate the risk factors for symptom numbers ≥ 3.

**Variables**	**Univariable ORs(95%CI)**	***P* value**	**Multivariable ORs(95%CI)**	***P* value**
Age, per year	1.03(1.02-1.03)	<0.001	1.02(1.01-1.03)	<0.001
Sex, vs. female	0.80(0.64-0.99)	0.04		
Severity, vs. no	1.97(1.57-2.47)	<0.001	1.51(1.14-1.99)	0.004
ICU admission, vs. no	1.90(1.02-3.53)	0.04		
Oxygen therapy, vs. no	1.54(1.19-2.00)	0.001		
Mechanical ventilation, vs. no	3.61(1.47-8.90)	0.005		
Cigarette smoking，vs. never				
Former	0.26(0.06-1.09)	0.07		
Active	0.98(0.63-1.52)	0.92		
Length of hospital stay, per day	1.02(1.00-1.03)	0.009		
Follow-up time, per day	0.98 (0.97-0.99)	<0.001	0.98(0.96-0.99)	<0.001
Coexisting disorder, vs. no				
Hypertension	1.17(0.92-1.48)	0.19		
Diabetes	1.27(0.94-1.71)	0.12		
Cardiovascular diseases	1.48(1.05-2.09)	0.02		
Chronic liver diseases	1.40(0.89-2.21)	0.15		
Cerebrovascular diseases	1.64(0.97-2.77)	0.06		

© 2021 Zhang X et al. *JAMA Network Open.*

Chronic kidney diseases	0.98(0.48-2.01)	0.96		
Tumour	1.59(0.78-3.24)	0.21		
COPD	1.90(0.74-4.84)	0.18		

Dependent variable: symptom numbers ≥ 3 v.s. symptom numbers < 3.

Independent variables: Age, Sex, Severity, ICU admission, Oxygen therapy (excluding mechanical ventilation), Mechanical ventilation, Cigarette smoking , Length of hospital stay, Follow-up time, Cardiovascular diseases, Cerebrovascular diseases.

Abbreviations: ICU, intensive care unit.

© 2021 Zhang X et al. *JAMA Network Open.*
